# Taguchi’s Orthogonal Arrays Are Classical Designs of Experiments

**DOI:** 10.6028/jres.096.034

**Published:** 1991

**Authors:** Raghu N. Kacker, Eric S. Lagergren, James J. Filliben

**Affiliations:** National Institute of Standards and Technology, Gaithersburg, MD 20899

**Keywords:** difference sets, fractional factorial plans, orthogonal arrays, Taguchi’s methods

## Abstract

Taguchi’s catalog of orthogonal arrays is based on the mathematical theory of factorial designs and difference sets developed by R. C. Bose and his associates. These arrays evolved as extensions of factorial designs and latin squares. This paper (1) describes the structure and constructions of Taguchi’s orthogonal arrays, (2) illustrates their fractional factorial nature, and (3) points out that Taguchi’s catalog can be expanded to include orthogonal arrays developed since 1960.

## 1. Introduction

Today many engineers are using Taguchi’s catalog of orthogonal arrays [1] to plan industrial experiments. But Taguchi provides either no information or insufficient information on the methods that were used to construct these arrays. Moreover, Taguchi displays orthogonal arrays in forms that are different from the way these arrays are usually displayed in the statistical literature. It is, therefore, difficult to discern the links between Taguchi’s arrays and their counterparts published elsewhere. Recent advertisements and testimonials of the efficacy of experiments based on Taguchi’s orthogonal arrays increase the confusion by giving an impression that these arrays are something other than fractional factorials and classical plans of experiments. This paper describes the structure and constructions of Taguchi’s orthogonal arrays, illustrates their fractional factorial nature, and points out that his catalog can be expanded to include orthogonal arrays developed since 1960.

The next section of this paper provides the background of orthogonal arrays and introduces the concept that an orthogonal array can be displayed in one of many equivalent forms. This concept is subsequently used to exhibit the equivalence of certain well-known fractional factorial plans and Taguchi’s orthogonal arrays. Taguchi’s catalog contains 20 arrays. However, only 18 of these arrays are orthogonal arrays. These 18 orthogonal arrays are the focus of this paper, and they have been classified into eight groups defined in such a way that the orthogonal arrays in each group can be constructed by a common method. The subsequent eight sections are devoted to these eight specific groups. In these sections, first the constructions of Taguchi’s orthogonal arrays are described and then these arrays are related to fractional factorials and other well-known orthogonal arrays. In order to appreciate the factorial nature of Taguchi’s orthogonal arrays, it is necessary to understand the constructions of these arrays. The last section of this paper identifies several useful orthogonal arrays that are not in Taguchi’s catalog because they were developed after 1960.

## 2. The Background of Orthogonal Arrays

An *orthogonal array* (more specifically a fixed-element orthogonal array) of *s* elements, denoted by OA_*N*_(*s^m^*) is an *N* × *m* matrix whose columns have the property that in every pair of columns each of the possible ordered pairs of elements appears the same number of times. The symbols used for the elements of an orthogonal array are arbitrary. This paper uses the symbols (0, 1, 2,…, *s* − 1) to denote the *s* elements. Tables 1 and 2 display OA_4_(2^3^) and OA_8_(2^7^) respectively. Note that in every pair of columns of table 1 each of the 4 ordered pairs (0,0), (0,1), (1,0), and (1,1) appears exactly once. Similarly, every pair of columns in table 2 contains each of the four pairs (0,0), (0,1), (1,0), and (1,1) exactly twice. Taguchi refers to *OA_N_*(_*s*_^*m*^) by the notation *L_N_*(*s^m^*). The letter L in this notation stands for latin square, and it indicates that orthogonal arrays are generalized latin squares. Taguchi uses the symbols (1,2,…,*s*) to denote the elements of an orthogonal array. The authors have, however, used the symbols (0,1,…, *s* − 1) in this paper because these symbols are natural in light of the methods of constructing these arrays.

Orthogonal arrays can be viewed as plans of multifactor experiments where the columns correspond to the factors, the entries in the columns correspond to the test levels of the factors and the rows correspond to the test runs. More specifically, the *N* rows of an OA_*N*_(*s^m^*) can be viewed as a subset of the possible *s^m^* test runs of a complete factorial plan in *m* factors each having *s* test levels. Thus, an OA_*N*_(*s^m^*) can be viewed as a *N/s^m^* fraction of a complete *s^m^* factorial plan. For example, the four rows of the OA_4_(2^3^) that are displayed in Table 1, can be viewed as a 4/2^3^ = 1/2 fraction of a complete 2^3^ factorial plan.

A sub-matrix formed by deleting some columns of an orthogonal array is also an orthogonal array. Thus, by deleting certain columns of a given orthogonal array, it is possible to generate many different plans of multifactor experiments.

A fractional factorial plan that enables uncorrelated estimation of every factorial effect included in the underlying linear model assuming that all other effects are zero is called an orthogonal plan. Fractional factorial plans based on orthogonal arrays irrespective of the degree of fractionation are necessarily orthogonal plans. This is the primary reason for the popularity of fractional factorials based on orthogonal arrays.

Some of the most popular arrays in Taguchi’s catalog are mixed-element (level) orthogonal arrays. A *mixed-element orthogonal army*, denoted by OA_*N*_(*s^m^ × t^n^*), is a matrix of *N* rows and *m + n* columns in which the first *m* columns have *s* elements each, the next *n* columns have *t* elements each, and in every pair of columns each of the possible ordered pairs of elements appears a constant number of times. The constant, however, depends on the pair of columns selected. Table 3 displays two orthogonal arrays: OA_18_(6 × 3^6^) and OA^18^(2 × 3^7^). Note that every pair of columns in Table 3 contains each of the possible ordered pairs a constant number of times. A mixed-level orthogonal array can be viewed as a fractionated multilevel factorial plan.

It follows from the definition of an orthogonal array that an orthogonal array remains an orthogonal array when its (1) rows are permuted or (2) columns are permuted or (3) the elements within a column are permuted. When orthogonal arrays are viewed as plans of multifactor experiments, the row permutation corresponds to reordering of test runs, the column permutation corresponds to relabeling of factors, and the permutation of elements within a column corresponds to relabeling of factor levels. Most experimenters realize that the labels of factors, the labels of factor levels and the order of test runs are arbitrary. Indeed, the order of test runs is usually randomized. Therefore, two orthogonal arrays are defined to be *equivalent* if one can be obtained from the other via the following permutations: (1) the rows are permuted, (2) the columns are permuted, and (3) the elements (symbols) within a column are permuted (for example, in a three-element column, the elements (0,1, and 2) can be replaced with any one of their permutations: (0,2, and 1); (1,0, and 2); (1,2 and 0); (2,0, and 1); or (2,1, and 0) respectively).

Taguchi’s format for an orthogonal array has the property that the entries in the left most columns change less frequently than the entries in the right most columns. Therefore, when these arrays are used to plan multifactor experiments, the cost of running the experiment can sometimes be reduced by judiciously associating with the left most columns those factors that are most expensive or most difficult to vary.

Taguchi’s catalog contains twenty arrays. However, only eighteen of these twenty arrays are orthogonal arrays. The remaining two arrays, denoted by L′_9_(2^21^) and L′_27_(3^22^), are not orthogonal arrays and they are not discussed in this paper. The eighteen orthogonal arrays are classified into eight groups based on the common method of construction. The next eight sections are devoted to these eight groups.

## 3. Two-Element Orthogonal Arrays of 2^*r*^ Rows for *r* = 2,3,4,5, and 6

The fractional factorial nature of two-element (level) orthogonal arrays follows from the way these arrays are constructed. So this section first describes a simple method of constructing these arrays, then illustrates their fractional factorial nature. A complete two-element orthogonal array with 2^*r*^ rows has 2^*r*^−1 columns and it is constructed column by column in three steps.

Step 1: Write in the *r* columns specified by column numbers 1,2,4,8,…, 2^*r*^^−1^ a complete factorial plan in *r* factors each having two test levels represented by 0 and 1 respectively. In order to match Taguchi’s display format, write this plan in such a way that the entries of the left most columns change less frequently than the entries of the right most columns. The entries of these *r* columns are used to calculate and define the entries of the remaining columns. Therefore, these *r* columns are referred to as the basic columns and marked as *x*_1_, *x*_2_,…, *x_r_*, respectively.
Step 2: These basic columns are used to generate the other columns. The generator of a particular column is a rule of the form *a*_1_*x*_1_ + *a*_2_*x*_2_ + … +*a_r_x_r_* where *x*_1_, *x*_2_,…,*x_r_* are the *r* basic columns and the coefficients *a*_1_, *a*_2_, …*a_r_* are obtained from the appropriate row of table 4. [For example, in the construction of OA_8_(2^7^) discussed below, the coefficients *a*_1_,*a*_2_,…, and *a*_3_ for column 5 are in the fifth row of table 4 and have the values 1, 0, and 1 respectively. This yields *x*_1_ + *x*_3_ as the generator for column 5 of OA_8_(2^7^)]. List the generators in the order of column numbers.
Step 3: Compute the entries of the other columns by using the generators identified in step 2. The required calculations are performed in modulo 2 arithmetic (that is, 0 + 0 = 0, 0+1 = 1, 1 + 0 = 1 and 1 + 1 = 0).

This method of construction and analogous methods of constructing three-, four-, and five-element orthogonal arrays are based on the mathematical theory of fractional factorials developed by Bose [2].

The following example illustrates these steps.

Example: Construction of an OA_8_(2^7^)

Here *N* = 8 = 2^3^, so *r* = 3.

Step 1: Write the *r* = 3 basic columns.

**Table:** 

Column No.	1	2	3	4	5	6	7
Row No.							
1	0			0			
2	0	0		1			
3	0	1		0			
4	0	1		1			
5	1	0		0			
6	1	0		1			
7	1	1		0			
8	1	1		1			

Generator	*x*_1_	*x*_2_		*x*_3_
Step 2: List the generators (see rows 1 to 7 of table 4).

**Table:** 

Column No.	Generator
1	*x*_1_
2	*x*_2_
3	*x*_1_ + *x*_2_
4	*x*_3_
5	*x*_1_ + *x*_3_
6	*x*_2_ + *x*_3_
7	*x*_1_ + *x*_2_ + *x*_3_
Step 3: Complete the array using the generators identified in step 2.

**Table:** 

Column No.	1	2	3	4	5	6	7
Row No.							
1	0	0	0	0	0	0	0
2	0	0	0	1	1	1	1
3	0	1	1	0	0	1	1
4	0	1	1	1	1	0	0
5	1	0	1	0	1	0	1
6	1	0	1	1	0	1	0
7	1	1	0	0	1	1	0
8	1	1	0	1	0	0	1

The fractional factorial nature of Taguchi’s two-element orthogonal arrays stems from the fact that the entries of the *r* columns identified by column numbers 1,2,4,8,…,2^*r*^^−1^ form a complete factorial plan, and the remaining columns correspond to the interaction effects. The generators of these columns have a one-to-one correspondence with the main effects and the interaction effects written in Yates’ [3] standard order. The *r* basic columns correspond to the main effects and the remaining columns correspond to the interaction effects.

A two-element (two-level) orthogonal array with 2^*r*^ rows reduces to a fractional factorial plan when more than *r* factors are associated with the columns of the array and the remaining columns are deleted. In particular, when all 2^*r*^^−1^ columns are associated with an equal number of factors, a two-level orthogonal array OA_*n*_(2^*m*^) where *N* = 2^*r*^ represents a *N/*2^*m*^ = (1/2)^*m*^^−^^*r*^ fraction of a complete 2^*m*^ factorial plan. For example, an OA_8_(2^7^) represents a (1/2)^7−3^ = (1/2)^4^ = 1/16th fraction of a complete 2^7^ factorial plan. That is, an OA_8_(2^7^) represents a 2^7−4^ plan in factorial notation.

The test levels of *2^k−p^* type fractional factorial plans are usually represented [4] by the symbols − and +. Such plans are often constructed by writing a complete factorial plan in the required number of test runs and appending additional columns obtained by multiplying certain columns of the complete factorial plan. This method and the Bose method of constructing two-level orthogonal arrays described here are similar, but since (− × − = +, − × + = −, and + × + = +) while (0 + 0 = 0, 0 + 1 = 1, and 1 + 1=0 in modulo 2 arithmetic), the two methods yield different fractions of the same type. However, one fraction can be obtained from another of the same type by switching the test levels, and permuting the rows and columns. For example, Taguchi’s OA_8_(2^7^) can be obtained from Box, Hunter, and Hunter’s 2^7−4^ plan [4] (shown as table 12.5 on page 391 of their book) by switching − and + in columns 4, 5 and 6; permuting the columns in the order 3,2,6,1,5,4, and 7; and relabeling − as 0 and + as 1.

## 4. Two-Element Orthogonal Array OA_12_(2^11^)

Table 5 displays Taguchi’s OA_12_(2^11^) in the 0 and 1 notation, and table 6 displays the classic Plackett and Burman [5] plan of 12 runs in the 0 and 1 notation rather than the usual − and + notation. Since table 5 can be obtained from table 6 through the following permutations, Taguchi’s OA_12_(2^11^) and the Plackett and Burman plan of 12 runs are equivalent.

1. In table 6 switch the elements 0 and 1 of columns 1,2,4,5,7, and 11 (that is, substitute 1 for 0 and 0 for 1 in these columns).
2. Permute the rows in the following order: 5,2,6,10,4,1,3,7,11,8,12,9.
3. Permute the columns in the following order: 1,2,3,4,6,5,9,10,8,7,11.

## 5. Three-Element Orthogonal Arrays of 3^*r*^ Rows for *r* = 2, 3, and 4

A complete three-element orthogonal array with 3^*r*^ rows has (3^*r*^− 1)/(3−1) columns and it is constructed in three steps:

Step 1: Write in the *r* columns specified by column numbers l,2,5,14,…,(3^*r*^^−1^−1)/(3−1) +1 a complete factorial plan in *r* factors each having three test levels represented by 0,1, and 2, respectively. In order to match Taguchi’s display format, write this plan in such a way that the entries of the left-most columns change less frequently than do the entries of the right-most columns. Mark these columns as *x*_1_, *x*_2_,…,*x^r^*, respectively.
Step 2: As before the generators of the remaining columns are of the form *a*_1_*x*_1_ + *a*_2_*x*_2_ +… + *a_r_x_r_* where *a*_1_*x*_1_,…,*x_r_* denote the *r* basic columns and the coefficients *a*_1_, *a*_2_,…, *a_r_* for a particular column are given in the appropriate row of table 7. List the generators in the order of column numbers.
Step 3: Compute the entries of the remaining columns by using the entries of the *r* basic columns and the appropriate generators. All calculations are done in modulo 3 arithmetic (that is, an integer larger than or equal to three is replaced with its remainder after division by three).

The following example illustrates these steps.

Example: Construction of an OA_9_(3^4^)

Here *N* = 9 = 3^2^, so *r* = 2.

Step 1: Write the *r* = 2 basic columns

**Table:** 

Column No.	1	2	3	4
Row No.				
1	0	0		
2	0	1		
3	0	2		
4	1	0		
5	1	1		
6	1	2		
7	2	0		
8	2	1		
9	2	2		

Generator	*x*_1_	*x*_2_
Step 2: List the generators (see rows 1 to 4 of table 7).

**Table:** 

Column No.	Generator
1	*x*_1_
2	*x*_2_
3	*x*_2_ + *x*_1_
4	*x*_2_ + 2*x*_1_
Step 3: Complete the array using the generators identified in step 2.

**Table:** 

Column No.	1	2	3	4
Row No.				
1	0	0	0	0
2	0	1	1	1
3	0	2	2	2
4	1	0	1	2
5	1	1	2	0
6	1	2	0	1
7	2	0	2	1
8	2	1	0	2
9	2	2	1	0

The fractional factorial nature of Taguchi’s three-element orthogonal arrays stems from the fact that the entries of the *r* basic columns identified by column numbers, 1,2,5,14,…,(3^*r*^^−1^−1)/(3−1) +1 form a complete factorial plan and the other columns correspond to the interaction effects. Since each column contains three distinct elements, two degrees of freedom are associated with each column. Since pairwise interaction effects carry (3−1)×(3−1) = 4 degrees of freedom, two columns correspond to each pairwise interaction effect. An interaction effect involving *k* factors carries (3−1)^*k*^ = 2^*k*^ degrees of freedom. Therefore, *2^k^*^−1^ columns correspond to each interaction effect involving *k* factors for *k* = 2,3,4,….

A three-element (level) orthogonal array with 3^*r*^ rows reduces to a fractional factorial plan when more than *r* factors are associated with the columns of the array and the remaining columns are deleted. In particular, when all (3^*r*^−1)/(3−1) columns are associated with an equal number of factors, a three-level orthogonal array OA_*N*_(3^*m*^) where *N* = 3^*r*^ and *m* = (3^*r*^−1)/(3 − 1) represents a *N/*3^*m*^ = (1/3)^*m−r*^ fraction of a complete 3^*m*^ factorial plan. For example, an OA_9_(3^4^) represents a (1/3)^2^ fraction of a complete 3^4^ factorial plan. That is, an OA_9_(3^4^) represents a 3^4−2^ plan in factorial notation.

## 6. Four-Element Orthogonal Arrays of 4^*r*^ Rows for *r* = 2 and 3

The method of constructing four-element orthogonal arrays is similar to the method for three-element arrays. An important difference, however, is that the calculations required to generate the columns are not performed in modulo 4 arithmetic. Instead, special addition and multiplication tables, displayed here as tables 9 and 10 are used. These addition and multiplication tables are based on the “finite arithmetic of the Galois Field Theory” that underlies this method of construction. According to this theory, the calculations required to generate an orthogonal array of *s* elements are done in modulo *s* arithmetic when *s* is a prime number, as is the case with 2, 3, and 5. When *s* is a power of a prime number such as 4 (which is the square of prime number 2), finite arithmetic of a Galois Field of *s* elements is used. A four-element orthogonal array with 4^*r*^ rows and (4^*r*^−1)/(4−1) columns is constructed in three steps.

Step 1: Write in the *r* columns specified by column numbers 1,2,6,…,(4^*r*^^−1^−1)/(4−1) +1 a complete factorial plan in *r* factors each having four test levels represented by 0,1,2, and 3, respectively. Write this plan in Taguchi’s format, and mark these columns as *x*_1_, *x*_2,…,_*x_r_*, respectively.
Step 2: As before the generators of the remaining columns are of the form *a*_1_*x*_1_ + *a*_2_*x*_2_ + … + *a_r_x_r_* where *x*_1_, *x*_2_,…,*x_r_* denote the *r* basic columns and the coefficients *a*_1_, *a*_2_,…, *a_r_* for a particular column are given in the appropriate row of table 8. List the generators in the order of column numbers.
Step 3: Compute the entries of the remaining columns by using the entries of the *r* basic columns and the appropriate generators. All calculations are done using finite additions and multiplications defined in tables 9 and 10.

The following example illustrates these steps.

Example: Construction of an OA_16_(4^5^)

Here *N* = 16 = 4^2^, so *r* = 2.

Step 1: Write the *r* = 2 basic columns

**Table:** 

Column No.	1	2	3	4	5
Row No.					
1	0	0			
2	0	1			
3	0	2			
4	0	3			
5	1	0			
6	1	1			
7	1	2			
8	1	3			
9	2	0			
10	2	1			
11	2	2			
12	2	3			
13	3	0			
14	3	1			
15	3	2			
16	3	3			

Generator	*x*_1_	*x*_2_
Step 2: List the generators (see rows 1 to 5 of table 8).

**Table:** 

Column No.	Generator
1	*x*_1_
2	*x*_2_
3	*x*_2_ + *x*_1_
4	*x*_2_ + 2*x*_1_
5	*x*_2_ + 3*x*_1_
Step 3: Complete the array using the generators identified in step 2 and finite additions and multiplications defined in tables 9 and 10.

**Table:** 

Column No.	1	2	3	4	5
Row No.					
1	0	0	0	0	0
2	0	1	1	1	1
3	0	2	2	2	2
4	0	3	3	3	3
5	1	0	1	2	3
6	1	1	0	3	2
7	1	2	3	0	1
8	1	3	2	1	0
9	2	0	2	3	1
10	2	1	3	2	0
11	2	2	0	1	3
12	2	3	1	0	2
13	3	0	3	1	2
14	3	1	2	0	3
15	3	2	1	3	0
16	3	3	0	2	1

The entries of the *r* basic columns identified by column numbers l,2,6,…,(4^*r*^^−1^−1)/(4−1) + 1 form a complete factorial plan and the other columns correspond to the interaction effects. Since each column contains four distinct elements, three degrees of freedom are associated with each column. An interaction effect involving *k* factors carries (4−1)^*k*^ = 3^*k*^ degrees of freedom. Therefore, 3^*k*^^−1^ columns correspond to each interaction effect involving *k* factors. In particular, three columns correspond to each pairwise interaction effect.

When all (4^*r*^−1)/(4−1) columns are associated with factors, a four-element (four-level) array OA_*N*_(4^*m*^) where *N* = 4^*r*^ and *m* = (4^*r*^−1)/(4−1) represents a *N*/4^*m*^ = (1/4)^*m*^^−^^*r*^ fraction of a complete 4^*m*^ factorial plan. For example, OA_16_(4^5^) represents a (1/4)^3^ fraction of a complete 4^5^ factorial plan.

## 7. Five-Element Orthogonal Array OA_25_(5^6^)

Taguchi’s OA_25_(5^6^) is constructed through the same general approach that is used to construct 2-, 3-, and 4-element arrays. The first two columns form a complete factorial plan in two factors each having five test levels represented by 0,1,2,3, and 4. The other columns are generated from these two columns using the following generators where *x*_1_ and *x*_2_ represent the entries of the first two columns.

**Table:** 

Column No.	Generator
1	*x*_1_
2	*x*_2_
3	*x*_2_ + *x*_1_
4	*x*_2_ + 2*x*_1_
5	*x*_2_ + 3*x*_1_
6	*x*_2_ + 4*x*_1_

All calculations are done in modulo 5 arithmetic. When the six columns of an OA_25_(5^6^) are associated with an equal number of factors, then OA_25_(5^6^) represents a (1/5)^4^ fraction of a complete 5^6^ factorial plan.

## 8. Mixed-element Orthogonal Arrays OA_18_(2^1^ × 3^7^), OA_32_(2^1^ × 4^9^) and OA_50_(2^1^ × 5^11^)

These orthogonal arrays are constructed by the method of Bose and Bush [6]. This method involves four concepts: difference matrices, Kronecker sums, saturated orthogonal arrays, and column replacement.

1. A *difference matrix* of *s* elements 0,1,…,(*s* − 1), denoted by *D_M_*(*s*), is an *M* × *M* matrix whose columns have the property that the differences in finite arithmetic between any two columns is a column in which each of the *s* elements occurs equally often. Table 11 displays the difference matrix D_3_(3). When *s* is a prime number such as 3 or 5, the finite arithmetic used in defining the difference matrix is modulo *s* arithmetic, and when *s* is a power of a prime number such as 4 (which is the square of prime number 2), the finite arithmetic is the arithmetic of a Galois Field of *s* elements. Table 9 defines finite addition for *s* = 4. The finite difference table for *s* = 4 is the same as the finite addition table.
2. The *Kronecker sum* of an *M* × *M* difference matrix D_*M*_(*s*) and a *p ×* 1 vector *b* of *s* elements, denoted by D_*M*_(*S*) ⨁*b*, is a matrix of *M* × *p* rows and *M* columns obtained by adding in finite arithmetic each element of the vector *b* to each element of the difference matrix D_*M*_(*S*). For example, if *b* is the column vector (0,1,2) and D_3_(3) is as defined in table 11, then
  $$D_{M}\left(s\right)⊕b=\left[\begin{array}{ccc} \begin{array}{c} 0+0\\ 0+1\\ 0+2 \end{array} & \begin{array}{c} 0+0\\ 0+1\\ 0+2 \end{array} & \begin{array}{c} 0+0\\ 0+1\\ 0+2 \end{array}\\ \begin{array}{c} \begin{array}{c} 0+0\\ 0+1 \end{array}\\ 0+2 \end{array} & \begin{array}{c} \begin{array}{c} 1+0\\ 1+1 \end{array}\\ 1+2 \end{array} & \begin{array}{c} \begin{array}{c} 2+0\\ 2+1 \end{array}\\ 2+2 \end{array}\\ \begin{array}{c} \begin{array}{c} 0+0 \end{array}\\ 0+1\\ 0+2 \end{array} & \begin{array}{c} \begin{array}{c} 2+0 \end{array}\\ 2+1\\ 2+2 \end{array} & \begin{array}{c} \begin{array}{c} 1+0 \end{array}\\ 1+1\\ 1+2 \end{array} \end{array}\right]=\left[\begin{array}{ccc} 0 & 0 & 0\\ 1 & 1 & 1\\ 2 & 2 & 2\\ 0 & 1 & 2\\ 1 & 2 & 0\\ 2 & 0 & 1\\ 0 & 2 & 1\\ 1 & 0 & 2\\ 2 & 1 & 0 \end{array}\right]$$
3. An orthogonal array OA_*N*_(*s^m^ × t^n^*) is said to be *saturated* when *N*−1 =* m*(*s* − 1)+*n*(t − 1). Since an *s* element column has *s − 1* degrees of freedom and a *t* element column has *t* − 1 degrees of freedom, *m +n* columns of OA_*N*_(*s^m^* × *t^n^*) have *m* (*s* − 1)+*n*(*t*−1) degrees of freedom. Since *the N* rows of OA_*N*_(*s^m^ × t^n^*) yield *N* (independent) data values, the total number of effects that can be estimated after allowing for the grand mean of the *N* data values is *N* − 1. Therefore when a saturated orthogonal array is used as an experimental plan, the total number of effects that can be estimated is equal to the total degrees of freedom of the columns (factors). When all *m +n* columns are associated with factors, a saturated orthogonal array can be viewed as a saturated main effect fractional factorial plan.
4. An orthogonal array remains an orthogonal array when one of its columns is replaced with an orthogonal array whose rows have a one-to-one correspondence with the elements of the replaced column. For example, suppose *a* is a four-element column of an orthogonal array *A*, and suppose *B* is an orthogonal array whose rows have a one-to-one correspondence with the elements of column *a* where
  $$a=\left[\begin{array}{c} 0\\ 1\\ 2\\ 3\\ 3\\ 2\\ 1\\ 0 \end{array}\right]\;\text{and}\;B=\left[\begin{array}{ccc} 0 & 0 & 0\\ 0 & 1 & 1\\ 1 & 0 & 1\\ 1 & 1 & 0\\ 1 & 1 & 0\\ 1 & 0 & 1\\ 0 & 1 & 1\\ 0 & 0 & 0 \end{array}\right]$$
  Then a matrix obtained from the orthogonal array *A* by replacing the column *a* with the orthogonal array *B* is an orthogonal array.

Taguchi’s OA_18_(2^1^ × 3^7^), OA_32_(2^1^ × 4^9^), and OA_50_(2^1^×5^11^) are constructed by the Bose and Bush method [6] from the difference matrices D_6_(3), D_8_(4), and D_10_(5) displayed in tables 12, 13, and 14 respectively. The following example illustrates the method.

Example: Construction of an OA_18_(2^1^ × 3^7^)

Step 1: Construct a matrix of 6 × 3 = 18 rows and 6 columns from the Kronecker sum of the difference matrix D_6_(3) displayed in table 12 and the column vector (0,1,2). This 18 × 6 matrix is displayed in columns 3,4,5,6,7, and 8 of table 3, and it is an OA_18_(3^6^).
Step 2: Append to columns 3,4,5,6,7, and 8 a six-element column consisting of three 0’s, three 1’s, three 2’s, three 3’s, three 4’s, and three 5’s. Label this column as 1′. Now columns 1′,3,4,5,6,7, and 8 of table 3 form a saturated orthogonal array OA_18_(6^1^ × 3^6^).
Step 3: Construct a matrix of 18 rows and 2 columns by associating the six ordered pairs (0,0), (0,1), (0,2), (1,0), (1,1), and (1,2) with the 6 elements 0,1,2,3,4, and 5 of column 1′. This matrix is an orthogonal array and its rows have a one-to-one correspondence with the elements of 1′. Write this orthogonal array in columns 1 and 2 of table 3. Now columns 1,2,3,4,5,6,7, and 8 of table 3 form Taguchi’s OA_18_(2^1^ × 3^7^).

Taguchi’s OA_32_(2^1^ × 4^9^) and OA_50_(2^1^ × 5^11^) are constructed, similarly, from the difference matrices D_8_(4) and D_10_(5) displayed in Tables 13 and 14, respectively.

The general method of constructing a mixed-element array of the type OA_*N*_(2^1^ × *s^m^*) where *m* = 2*s* + 1 and *N* = 2*s*^2^ from a difference matrix of the type *D*_2__*s*_ (*s*) where *s* is a prime number (such as 3 or 5) or a power of a prime number (such as 4) consists of three steps.

Step 1: Construct a matrix of 2*s*^2^ rows and 2*s* columns from the Kronecker sum of the difference matrix *D*_2__*s*_(*s*) and the column vector (0,1,…, *s* − 1). These 2*s* columns form OA_*N*_(*s*^2^^*s*^) where *N* = 2*s*^2^. Label these columns as 3,4,…, and 2*s +* 2, respectively.
Step 2: Append a 2*s* -element column consisting of *s* 0’s, *s* 1’s, …, and *s* (2*s* − 1)’s. (The total number of entries in the column is 2*s × s* = 2*s*^2^). Label this column 1*′.* Now columns 1′,3,4,…, and 2*s +*2 form a saturated orthogonal array OA_*N*_[(2*S*)^1^ × *S*^2^_*S*_] where *N* = 2*s*^2^.
Step 3: Construct a matrix of 2*s*^2^ rows and 2 columns by associating the 2*s* ordered pairs (0,0), (0,1),…, (0,*s*−1), (1,0), (1,1),…, and (1,*s*−1) with the 2*s* elements 0,1,…, *s* − 1, *s*, *s* + 1,…, and 2*s* − 1 of column 1′. This matrix is an orthogonal array and its rows have a one-to-one correspondence with the elements of column 1′. Write this orthogonal array in columns 1 and 2. Now columns 1,2,3,…, 2*s* + 2 form OA_*N*_(2^1^ × *s^m^*) where *N* = 2*s*^2^ and *m* =25 + 1.

A difference matrix remains a difference matrix when (1) its rows are permuted or (2) its columns are permuted or (3) an integer is added (in finite arithmetic) to any column of the matrix. Because of finite arithmetic, the addition of an integer to a column results in a permutation of the elements of that column. Thus each of these three operations results in a permutation of the elements of the matrix. The difference matrices D_6_(3) and Ds(4) used by Taguchi are permuted versions of Bose and Bush’s D_6_(3) and D_8_(4), respectively, and Taguchi’s D_10_(5) is a permuted version of Masuyama’s [7] D_10_(5). Specifically, Taguchi’s D_6_(3) displayed in table 12 can be obtained from Bose and Bush’s D_6_(3) displayed in table 15 by permuting the columns of table 15 in the following order: 1,2,3,5,6, and 4. And Taguchi’s D_8_(4) displayed in table 13 can be obtained from Bose and Bush’s D_8_(4) displayed in table 16 by permuting the columns of table 16 in the following order: 1,5,2,6,3,7,4, and 8. Similarly, Taguchi’s D_10_(5) displayed in table 14 can be obtained from Masuyama’s D_10_(5) displayed in table 17 by permuting both the rows and the columns of table 17 in the following order: 1,2,3,4,5,10,6,7,8, and 9.

When all *m* + 1 columns are associated with factors, a mixed-element (mixed-level) orthogonal array, OA_*N*_(2^1^ × *s^m^*), where *N* = 2*s*^2^ and *m*= 2*s* + 1 represents a *N*/(2^1^ × *s^m^*) = (1/*s*)^*m*^^−2^ fractional factorial plan. For example, OA_18_(2^1^ × 3^7^) can be viewed as a (1/3)^7−2^ = (1/3)^5^ fraction of a complete 2^1^ × 3^7^ factorial plan.

## 9. Mixed-Element Orthogonal Arrays OA_36_(2^11^ × 3^12^) and OA_36_(2^3^ × 3^13^)

These arrays are constructed by appending certain columns to OA_36_(3^12^) developed by the Bose and Bush method [6] from the difference matrix D_12_(3) displayed in table 19. The following steps describe the method.

Step 1: Construct a matrix of 12 × 3 = 36 rows and 12 columns from the Kronecker sum of the difference matrix D_12_(3) displayed in table 19 and the column vector (0,1,2). These twelve columns are displayed in columns 12,13,…, and 23 of table 18 and they form OA_36_(3^12^).
Step 2: Append to columns 12,13,…, and 23 a twelve-element column consisting of three 0’s, three 1s,…, and three 11’s. Label this column as 1″. Now columns 12,13,…, 22, 23, and 1″ form a saturated orthogonal array OA_36_(3^12^ × 12^1^).
Step 3: Construct a matrix of 12 × 3 = 36 rows and 11 columns by repeating three times each row of OA_12_(2^11^) displayed in table 5. This matrix is an orthogonal array and its rows have a one-to-one correspondence with the twelve elements of column 1”. Write this orthogonal array in columns 1,2,…,10, and 11. Now columns 1,2,…,11,12,13,…, and 23 of table 18 form Taguchi’s OA_36_(2^11^ × 3^12^).
Step 4: Construct a matrix of 12 rows and 4 columns by repeating OA_4_(2^3^) displayed in table 1 three times and appending a three-element column consisting of four O’s, four 1’s and four 2’s. These four columns form OA_12_(2^3^ × 3^1^), and are displayed in table 20. Now construct a matrix of 36 rows and 4 columns by repeating three times each of the twelve rows of OA_12_(2^3^ × 3^1^) displayed in table 20. This 36 × 4 matrix is an orthogonal array of 36 rows and 4 columns and its rows have a one-to-one correspondence with the elements of column 1″. Write this orthogonal array in columns l′,2′,3′, and 4′ of table 18. Now columns 12,13,…, 23, l′,2′,3′, and 4′ of table 18 form Taguchi’s OA_3_6(2^3^ × 3^13^).

The difference matrix D_12_(3) used by Taguchi is a permuted version of Seiden’s [8] D_12_(3). Specifically, Taguchi’s D_12_(3) displayed in table 19 can be obtained from Seiden’s D_12_(3) displayed in table 21 through two operations. First add 1 (in modulo 3 arithmetic) to each element of columns 10 and 11, and add 2 to each element of columns 4,5, and 8 in table 21, then permute the columns in the following order: 1,2,3,6,12,10,11,5,9,7,8, and 4.

When all columns of OA_3_6(2^11^ × 3^12^) are associated with factors, this array represents a 36/(2^11^ × 3^12^) fraction of a complete 2^11^ × 3^12^ factorial plan; a very highly fractionated orthogonal plan indeed. Similarly OA_36_,(2^3^ × 3^13^) represents a 36/(2^3^ × 3^13^) fraction of a complete 2^3^ × 3^13^ factorial plan.

## 10. Mixed-Element Orthogonal Array OA_54_(2^1^ × 3^25^)

This array is a special case of OA_54_(6^1^ × 3^24^) where the six-element column is replaced with two columns one having two elements and the other having three elements. Orthogonal array OA_54_(6^1^ × 3^24^) is displayed in table 22 in a special vector form (to save space). In table 22 boldface numbers and letters **0,1,2,3,4,5,*a*,*b***, and c represent the column vectors (0,0,0), (1,1,1), (2,2,2), (3,3,3), (4,4,4), (5,5,5), (0,1,2), (1,2,0), and (2,0,1), respectively. An OA_54_(6^1^ × 3^24^) is constructed from OA_18_(6^l^ × 3^6^), displayed in table 3, in three steps. (1) Repeat three times each row of OA_18_(6^1^ × 3^6^). (2) Append a column consisting of the vector (0,1,2) repeated eighteen times. (3) Append seventeen additional columns representing the interactions of the columns of OA_18_(6^1^ × 3^6^) and the three-element column appended in step 2. The following paragraphs describe these steps in more detail.

Step 1: Construct a 54 × 7 matrix by repeating three times each row of OA_18_(6^1^ × 3^6^) displayed in table 3. This matrix forms the columns 1′,3,4,5,6,7, and 8 of table 22.
Step 2: Append to columns 1′,3,4,5,6,7, and 8 of table 22, a three-element column consisting of the vector (0,1,2) repeated eighteen times. This is column 9 of table 22.
Step 3: The seventeen columns identified by column numbers 10,11,12,…,25 and 26 of table 22 represent the interactions of column 9 with each of the seven columns 1′,3,4,5,6,7, and 8. Since column 1*′* has 6 elements (5 degrees of freedom) and column 9 has 3 elements (2 degrees of freedom), interaction of column *V* and column 9 has 5 × 2 = 10 degrees of freedom. Thus 5 three-element columns (2 degrees of freedom each) are needed to represent the interaction of column 1*′* and column 9. These five columns are constructed from columns 2,3,4,5, and 6 of the difference matrix D_6_(3) displayed in table 12 as follows. Construct an 18 × 5 matrix (not shown in this paper) by deleting column 1 of table 12 and repeating three times each row of the remaining five columns. Now construct a matrix of 54 rows and 5 columns by the Kronecker sum of this 18 × 5 matrix and the column vector (0,1,2). These five columns are displayed in columns 10,11,12,13, and 14 of table 22 and they represent the interaction of column 1*′* and column 9.

Since each of the columns 3,4,5,6,7,8, and 9 has three elements (2 degrees of freedom), each pair-wise interaction among these columns has 4 degrees of freedom. Thus 2 three-element columns (2 degrees of freedom each) are needed to represent each pairwise interaction of column 9 with the columns 3,4,5,6,7, and 8. These interaction columns are identified by the method of Bose [2]. This method of construction was used earlier in this paper to construct 2-, 3-, 4-, and 5- element orthogonal arrays. Mark columns 3,4,5,6,7,8, and 9 as *x*_3_, *x*_4_, *x*_5_, *x*_6_, *x*_7_, *x*_8_, and *x*_9_, respectively. These seven columns are used to generate the columns 15,16,17,18,19,20,21,22,23,24,25, and 26 using the following generators, respectively.

**Table:** 

Column No.	Generator
15	*x*_9_ *+ x*_3_
16	*x*_9_ *+* 2*x*_3_
17	*x*_9_ *+ x*_4_
18	*x*_9_ *+* 2*x*_4_
19	*x*_9_ *+ x*_5_
20	*x*_9_ *+* 2*x*_5_
21	*x*_9_ *+ x*_6_
22	*x*_9_ *+* 2*x*_6_
23	*x*_9_ *+ x*_7_
24	*x*_9_ *+* 2*x*_7_
25	*x*_9_ *+ x*_8_
26	*x*_9_ *+* 2*x*_8_

All calculations are done in modulo 3 arithmetic. Note that columns 15 and 16 together represent the four degrees of freedom corresponding to the interaction of column 3 and column 9. Similarly the other columns (in pairs) represent the interactions involving column 9, and columns 4,5,6,7, and 8, respectively. Now columns 1′,3,4,5,…,25, and 26 of table 22 form OA_54_(6^1^ × 3^24^).

Taguchi’s OA_54_(2^1^ × 3^25^) is constructed by replacing column 1′ with two columns formed by associating the 6 ordered pairs (0, 0),(0, 1),(0, 2), (1, 0),(1, 1), and (1, 2) with the elements 0,1,2,3,4, and 5 of column 1′. These two columns are displayed in columns 1 and 2 of table 22. Now columns 1,2,3,4,…, 25 and 26 of table 22 form Taguchi’s OA_54_(2^1^ × 3^25^). When all columns of OA_54_(2^1^ × 3^25^) are associated with factors, this array represents a 54/(2^1^ × 3^25^) fraction of a complete 2^1^ × 3^25^ factorial plan.

## 11. Concluding Remarks

As shown in this paper, Taguchi’s catalog of orthogonal arrays [1] is primarily based on two papers: Bose [2], and Bose and Bush [6]. The Bose paper laid the mathematical foundation of fractional factorials and orthogonal arrays. The Bose and Bush paper describes a method of constructing mixed-element orthogonal arrays from difference matrices. The difference matrices used by Taguchi to construct O_A18_(2^1^ × 3^7^), OA_32_(2^1^ × 4^9^), OA_36t_(2^11^ × 3^12^),OA_36_(2^3^ × 3^13^)and OA_50_(2^1^ × 5^11^) are permuted versions of the difference matrices developed by Bose and Bush [6], Seiden [8], and Masuyama [7]. The extensions of the Bose, and the Bose and Bush, methods needed to construct OA_36_(2^11^ × 3^12^),OA_36_(2^3^ × 3^13^), and OA_54_(2^1^ × 3^25^) appear to be Taguchi’s contributions. The authors have shown that Taguchi’s OA_12_(2^11^) is a permuted version of the Plackett and Burman [5] plan of 12 runs, but there is no reason to believe that Taguchi permuted the Plackett and Burman plan to construct his OA_12_(2^11^).

Although the original Japanese version of Taguchi’s catalog of orthogonal arrays was developed before 1960, it continues to be very useful. These arrays can be modified to generate many types of multifactor experiments [9] and many other orthogonal arrays can be derived from Taguchi’s catalog through established mathematical procedures. Nevertheless, the catalog can now be expanded to include arrays developed after 1960. For example, Taguchi’s catalog can be expanded to include OA_24_(4^1^ × 2^20^), OA_40_(4^1^ × 2^36^) and OA_48_(4^3^ × 2^38^) first developed by Dey and Ramakrishna [10] and Chacko, Dey, and Ramakrishna [11], and then re-constructed through a unified procedure by Cheng [12]. It is the authors’ intent to develop an expanded and revised version of Taguchi’s catalog of orthogonal arrays. A companion paper [13] limited to the fixed-element orthogonal arrays appeared in the Journal of Quality Technology.

## Figures and Tables

**Table 1:** Orthogonal array OA_4_ (2^3^)

Row No.	Column No.		
	1	2	3
1	0	0	0
2	0	1	1
3	1	0	1
4	1	1	0

**Table 2:** Orthogonal array OA_8_ (2^7^)

Row No.	Column No.						
	1	2	3	4	5	6	7
1	0	0	0	0	0	0	0
2	0	0	0	1	1	1	1
3	0	1	1	0	0	1	1
4	0	1	1	1	1	0	0
5	1	0	1	0	1	0	1
6	1	0	1	1	0	1	0
7	1	1	0	0	1	1	0
8	1	1	0	1	0	0	1

**Table 3:** Orthogonal arrays OA_18_ (6^1^ × 3^6^) and OA_18_ (2^1^ × 3^7^)^a^

Row No.	Column No.								
	1*′*	1	2	3	4	5	6	7	8
1	0	0	0	0	0	0	0	0	0
2	0	0	0	1	1	1	1	1	1
3	0	0	0	2	2	2	2	2	2
4	1	0	1	0	0	1	1	2	2
5	1	0	1	1	1	2	2	0	0
6	1	0	1	2	2	0	0	1	1
7	2	0	2	0	1	0	2	1	2
8	2	0	2	1	2	1	0	2	0
9	2	0	2	2	0	2	1	0	1
10	3	1	0	0	2	2	1	1	0
11	3	1	0	1	0	0	2	2	1
12	3	1	0	2	1	1	0	0	2
13	4	1	1	0	1	2	0	2	1
14	4	1	1	1	2	0	1	0	2
15	4	1	1	2	0	1	2	1	0
16	5	1	2	0	2	1	2	0	1
17	5	1	2	1	0	2	0	1	2
18	5	1	2	2	1	0	1	2	0

a Columns 1′,3,4,5,6,7, and 8 form OA_18_(6^1^ × 3^6^).

Columns 1,2,3,4,5,6,7, and 8 form OA_18_(2^1^ × 3^7^).

**Table 4:** Coefficients of the generators of two-element orthogonal arrays of 2′ rows for *r* = 2, 3,…

Column No.	Coefficients of *x*_1_, *x*_2_, *x*_3_,…,*x_r_*					
	*a*_1_	*a*_2_	*a*_3_	…	*a_r_*−1	*a_r_*
1	1	0	0	First (2^*r*^^−2^−1) entries are 0		First (2^r−1^−1) entries are 0
2	0	1	0			
3	1	1	0	Next (2^*r*^^−2^) entries are 1		Last (2^*r*^^−1^) entries are 1
4	0	0	1			
5	1	0	1	Next (2^*r*^^−2^) entries are 0		
6	0	1	1			
7	1	1	1	Last (2^*r*^^−2^) entries are 1		
·	Repeat	Repeat				
·	(0,1)	(0,0,1,1)				
2^*r*^−2	0	1	1			
2^*r*^−1	1	1	1			

**Table 5:** Orthogonal array OA_12_(2^11^)

Row No.	Column No.										
	1	2	3	4	5	6	7	8	9	10	11
1	0	0	0	0	0	0	0	0	0	0	0
2	0	0	0	0	0	1	1	1	1	1	1
3	0	0	1	1	1	0	0	0	1	1	1
4	0	1	0	1	1	0	1	1	0	0	1
5	0	1	1	0	1	1	0	1	0	1	0
6	0	1	1	1	0	1	1	0	1	0	0
7	1	0	1	1	0	0	1	1	0	1	0
8	1	0	1	0	1	1	1	0	0	0	1
9	1	0	0	1	1	1	0	1	1	0	0
10	1	1	1	0	0	0	0	1	1	0	1
11	1	1	0	1	0	1	0	0	0	1	1
12	1	1	0	0	1	0	1	0	1	1	0

**Table 6:** Plackett and Burman plan of 12 rows

Row No.	Column No.										
	1	2	3	4	5	6	7	8	9	10	11
1	1	0	1	0	0	0	1	1	1	0	1
2	1	1	0	1	0	0	0	1	1	1	0
3	0	1	1	0	1	0	0	0	1	1	1
4	1	0	1	1	0	1	0	0	0	1	1
5	1	1	0	1	1	0	1	0	0	0	1
6	1	1	1	0	1	1	0	1	0	0	0
7	0	1	1	1	0	1	1	0	1	0	0
8	0	0	1	1	1	0	1	1	0	1	0
9	0	0	0	1	1	1	0	1	1	0	1
10	1	0	0	0	1	1	1	0	1	1	0
11	0	1	0	0	0	1	1	1	0	1	1
12	0	0	0	0	0	0	0	0	0	0	0

**Table 7:** Coefficients of the generators of three-element orthogonal arrays of 3^*r*^ rows for *r* = 2, 3,…

Column No.	Coeffieicnts of *x*_1_, *x*_2_, *x*_3_,…,*x_r_*				
	*a*_1_	*a*_2_	…	*a_r_*_−1_	*a_r_*
1	1	0	First (3^*r*^^−1^−1)/(3−1) entries arc 0		First(3^*r*^^−1^−1)/(3−1) entries arc 0
2	0	1			
3	1	1	Next (3^*r*^^−2^) entries are 1		Last (3^*r*^^−1^) entries are 1
4	2	1			
	Repeat	Repeat	Next (3^*r*^^−2^) entries are 0		
·	(0,1,2)	(0,0,0, 1,1,1, 2,2,2)			
·			Next(3^*r*^^−2^) entries are 1		
·					
·			Last (3^*r*^^−2^) entries are 2		
·					
(3^*r*^−1)/(3−1) 2	2	2			

**Table 8:** Coefficients of the generators of four-element orthogonal arrays of 4^*r*^ rows for *r* = 2, 3,…

Column No.	Coefficients of *x*_1_*x*_2_,…,*x_r_*				
	*a*_1_	*a*_2_	…	*a_r_*_−1_	*a_r_*
1	1	0	First (4^*r*^^−2^−1)/(4−1) entries are 0		First (4^*r*^^−2^−1)/(4−1) entries are 0
2	0	1			
3	1	1	Next (4^*r*^^−2^) entries are 1		Last (4^*r*^^−1^) entries are 1
4	2	1			
5	3	1	Next (4^*r*^^−2^) entries are 0		
·	Repeat (0,1,2,3)				
·			Next (4^*r*^^−2^) entries are 1		
·					
·			Next (4^*r*^^−2^) entries are 2		
·					
·			Last (4^*r*^^−2^) entries are 3		
·					
·					
·					
(4^*r*^^−2^)/(4−1)	3	3			

**Table 9:** Addition table for a Galois field of four elements^a^

+	0	1	2	3
0	0	1	2	3
1	1	0	3	2
2	2	3	0	1
3	3	2	1	0

a This addition table is also the difference table for a Galois field of four elements.

**Table 10:** Multiplication table for a Galois field of four elements

×	0	1	2	3
0	0	0	0	0
1	0	1	2	3
2	0	2	3	1
3	0	3	1	2

**Table 11:** Difference matrix D_3_(3)

Row No.	Column No.		
	1	2	3
1	0	0	0
2	0	1	2
3	0	2	1

**Table 12:** Taguchi’s difference matrix D_6_(3)

Row No.	Column No.					
	1	2	3	4	5	6
1	0	0	0	0	0	0
2	0	0	1	1	2	2
3	0	1	0	2	1	2
4	0	2	2	1	1	0
5	0	1	2	0	2	1
6	0	2	1	2	0	1

**Table 13:** Taguchi’s difference matrix D_8_(4)

Row No.	Column No.							
	1	2	3	4	5	6	7	8
1	0	0	0	0	0	0	0	0
2	0	0	1	1	2	2	3	3
3	0	1	2	3	0	1	2	3
4	0	1	3	2	2	3	1	0
5	0	3	0	3	1	2	1	2
6	0	3	1	2	3	0	2	1
7	0	2	2	0	1	3	3	1
8	0	2	3	1	3	1	0	2

**Table 14:** Taguchi’s difference matrix D_10_(5)

Row No.	Column No.									
	1	2	3	4	5	6	7	8	9	10
1	0	0	0	0	0	0	0	0	0	0
2	0	1	2	3	4	0	1	2	3	4
3	0	2	4	1	3	3	0	2	4	1
4	0	3	1	4	2	4	2	0	3	1
5	0	4	3	2	1	3	2	1	0	4
6	0	0	3	4	3	2	1	4	1	2
7	0	1	0	2	2	1	3	4	4	3
8	0	2	2	0	1	4	4	3	1	3
9	0	3	4	3	0	1	4	1	2	2
10	0	4	1	1	4	2	3	3	2	0

**Table 15:** Bose and Bush’s difference matrix D_6_(3)

Row No.	Column No.					
	1	2	3	4	5	6
1	0	0	0	0	0	0
2	0	0	1	2	1	2
3	0	1	0	2	2	1
4	0	2	2	0	1	1
5	0	1	2	1	0	2
6	0	2	1	1	2	0

**Table 16:** Bose and Bush’s difference matrix D_8_(4)

Row No.	Column No.							
	1	2	3	4	5	6	7	8
1	0	0	0	0	0	0	0	0
2	0	1	2	3	0	1	2	3
3	0	2	0	2	1	3	1	3
4	0	3	2	1	1	2	3	0
5	0	0	1	1	3	3	2	2
6	0	1	3	2	3	2	0	1
7	0	2	1	3	2	0	3	1
8	0	3	3	0	2	1	1	2

**Table 17:** Masuyama’s difference matrix D_10_(5)

Row No.	Column No.									
	1	2	3	4	5	6	7	8	9	10
1	0	0	0	0	0	0	0	0	0	0
2	0	1	2	3	4	1	2	3	4	0
3	0	2	4	1	3	0	2	4	1	3
4	0	3	1	4	2	0	0	3	1	4
5	0	*4*	3	2	1	2	1	0	4	3
6	0	1	0	2	2	3	4	4	3	1
7	0	2	2	0	1	4	3	1	3	4
8	0	3	4	3	0	4	1	2	2	1
9	0	4	1	1	4	3	3	2	0	2
10	0	0	3	4	3	1	4	1	2	2

**Table 18:** Orthogonal arrays OA_36_(2^11^ × 3^12^), OA_36_(2^3^ × 3^13^), and OA_36_(3^12^ × 12^1^)^a^

Row No.	Column No.																											
	1	2	3	4	5	6	7	8	9	10	11	12	13	14	15	16	17	18	19	20	21	22	23	1′	2′	3	4′	1″
1	0	0	0	0	0	0	0	0	0	0	0	0	0	0	0	0	0	0	0	0	0	0	0	0	0	0	0	0
2	0	0	0	0	0	0	0	0	0	0	0	1	1	1	1	1	1	1	1	1	1	1	1	0	0	0	0	0
3	0	0	0	0	0	0	0	0	0	0	0	2	2	2	2	2	2	2	2	2	2	2	2	0	0	0	0	0
4	0	0	0	0	0	1	1	1	1	1	1	0	0	0	0	1	1	1	I	2	2	2	2	0	1	1	0	1
5	0	0	0	0	0	1	1	1	1	1	1	1	1	1	1	2	2	2	2	0	0	0	0	0	1	1	0	1
6	0	0	0	0	0	1	1	1	1	1	1	2	2	2	2	0	0	0	0	1	1	1	1	0	1	1	0	1
7	0	0	1	1	1	0	0	0	1	1	1	0	0	1	2	0	1	2	2	0	1	1	2	1	0	1	0	2
8	0	0	1	1	1	0	0	0	1	1	1	1	1	2	0	1	2	0	0	1	2	2	0	1	0	1	0	2
9	0	0	1	1	1	0	0	0	1	1	1	2	2	0	1	2	0	1	1	2	0	0	1	1	0	1	0	2
10	0	1	0	1	1	0	1	1	0	0	1	0	0	2	1	0	2	1	2	1	0	2	1	1	1	0	0	3
11	0	1	0	1	1	0	1	1	0	0	1	1	1	0	2	1	0	2	0	2	1	0	2	1	1	0	0	3
12	0	1	0	1	1	0	1	1	0	0	1	2	2	1	0	2	1	0	1	0	2	1	0	1	1	0	0	3
13	0	1	1	0	1	1	0	1	0	1	0	0	1	2	0	2	1	0	2	2	1	0	1	0	0	0	1	4
14	0	1	1	0	1	1	0	1	0	1	0	1	2	0	1	0	2	1	0	0	2	1	2	0	0	0	1	4
15	0	1	1	0	1	1	0	1	0	1	0	2	0	1	2	1	0	2	1	I	0	2	0	0	0	0	1	4
16	0	1	1	1	0	1	1	0	1	0	0	0	1	2	1	0	0	2	1	2	2	1	0	0	1	1	1	5
17	0	1	1	1	0	1	1	0	1	0	0	1	2	0	2	1	1	0	2	0	0	2	1	0	1	1	1	5
18	0	1	1	1	0	1	1	0	1	0	0	2	0	1	0	2	2	1	0	1	I	0	2	0	1	1	1	5
19	1	0	1	1	0	0	1	1	0	1	0	0	1	0	2	2	2	0	1	1	0	1	2	1	0	1	1	6
20	1	0	1	1	0	0	1	1	0	1	0	1	2	1	0	0	0	1	2	2	1	2	0	1	0	1	1	6
21	1	0	1	1	0	0	1	1	0	1	0	2	0	2	1	1	1	2	0	0	2	0	1	1	0	1	1	6
22	1	0	1	0	1	1	1	0	0	0	1	0	1	1	2	2	0	1	0	0	2	2	1	1	1	0	1	7
23	1	0	1	0	1	1	1	0	0	0	1	1	2	2	0	0	1	2	1	1	0	0	2	1	1	0	1	7
24	1	0	1	0	1	1	1	0	0	0	1	2	0	0	1	1	2	0	2	2	1	1	0	1	1	0	1	7
25	1	0	0	1	1	1	0	1	1	0	0	0	2	1	0	1	2	2	0	2	0	1	1	0	0	0	2	8
26	1	0	0	1	1	1	0	1	1	0	0	1	0	2	1	2	0	0	1	0	1	2	2	0	0	0	2	8
27	1	0	0	1	1	1	0	1	1	0	0	2	1	0	2	0	1	1	2	1	2	0	0	0	0	0	2	8
28	1	1	1	0	0	0	0	1	1	0	1	0	2	1	1	1	0	0	2	1	2	0	2	0	1	1	2	9
29	1	1	1	0	0	0	0	1	1	0	1	1	0	2	2	2	1	1	0	2	0	1	0	0	1	1	2	9
30	1	1	1	0	0	0	0	1	1	0	1	2	1	0	0	0	2	2	1	0	1	2	1	0	1	1	2	9
31	1	1	0	1	0	1	0	0	0	1	1	0	2	2	2	1	2	1	1	0	1	0	0	1	0	1	2	10
32	1	1	0	1	0	1	0	0	0	1	1	1	0	0	0	2	0	2	2	1	2	1	1	1	0	1	2	10
33	1	1	0	1	0	1	0	0	0	1	1	2	1	1	1	0	1	0	0	2	0	2	2	1	0	1	2	10
34	1	1	0	0	1	0	1	0	1	1	0	0	2	0	1	2	1	2	0	1	1	2	0	1	1	0	2	11
35	1	1	0	0	1	0	1	0	1	1	0	1	0	1	2	0	2	0	1	2	2	0	1	1	1	0	2	11
36	1	1	0	0	1	0	1	0	1	1	0	2	1	2	0	1	0	1	2	0	0	1	2	1	1	0	2	11

a Columns 1,2,…,22, and 23 form OA_36_(2^11^ × 3^12^).

Columns 1″,12,13,…,22, and 23 form OA_36_(3^12^ × 12^1^).

Columns 1′,2′ 3′,4′,12,13,…,22 and 23 form OA_36_(2^3^ × 3^13^).

**Table 19:** Taguchi’s difference matrix D_12_(3)

Row No.	Column No.											
	1	2	3	4	5	6	7	8	9	10	11	12
1	0	0	0	0	0	0	0	0	0	0	0	0
2	0	0	0	0	1	1	1	1	2	2	2	2
3	0	0	1	2	0	1	2	2	0	1	1	2
4	0	0	2	1	0	2	1	2	1	0	2	1
5	0	1	2	0	2	1	0	2	2	1	0	1
6	0	1	2	1	0	0	2	1	2	2	1	0
7	0	1	0	2	2	2	0	1	1	0	1	2
8	0	1	1	2	2	0	1	0	0	2	2	1
9	0	2	1	0	1	2	2	0	2	0	1	1
10	0	2	1	1	1	0	0	2	1	2	0	2
11	0	2	2	2	1	2	1	1	0	1	0	0
12	0	2	0	1	2	1	2	0	1	1	2	0

**Table 20:** Orthogonal array OA_12_(2^3^ × 3^1^)

Row No.	Column No.			
	1	2	3	4
1	0	0	0	0
2	0	1	1	0
3	1	0	1	0
4	1	1	0	0
5	0	0	0	1
6	0	1	1	1
7	1	0	1	1
8	1	1	0	1
9	0	0	0	2
10	0	1	1	2
11	1	0	1	2
12	1	1	0	2

**Table 21:** Seiden’s difference matrix D_12_(3)

Row No.	Column No.											
	1	2	3	4	5	6	7	8	9	10	11	12
1	0	0	0	1	1	0	0	1	0	2	2	0
2	0	0	0	0	2	0	2	0	2	0	0	1
3	0	0	1	0	0	2	1	2	0	0	1	0
4	0	0	2	2	0	1	0	0	1	1	0	0
5	0	1	2	2	0	0	1	1	2	0	2	2
6	0	1	2	1	2	1	2	2	2	2	1	0
7	0	1	0	0	2	2	0	2	1	1	2	2
8	0	1	1	2	1	2	2	0	0	2	0	2
9	0	2	1	2	1	0	0	2	2	1	1	1
10	0	2	1	0	0	1	2	1	1	2	2	1
11	0	2	2	1	2	2	1	1	0	1	0	1
12	0	2	0	1	1	1	1	0	1	0	1	2

**Table 22:** Orthogonal array OA_54_(2^1^ × 3^25^), and OA_54_ (6^1^ × 3^24^)^a^

Row No.	Column No.																										
	1′	1	2	3	4	5	6	7	8	9	10	11	12	13	14	15	16	17	18	19	20	21	22	23	24	25	26
1–3	0	0	0	0	0	0	0	0	0	*a*	*a*	*a*	*a*	*a*	*a*	*a*	*a*	*a*	*a*	*a*	*a*	*a*	*a*	*a*	*a*	*a*	*a*
4–6	0	0	0	1	1	1	1	1	1	*a*	*a*	*a*	*a*	*a*	*a*	*b*	*c*	*b*	*c*	*b*	*c*	*b*	*c*	*b*	*c*	*b*	*c*
7–9	0	0	0	2	2	2	2	2	2	*a*	*a*	*a*	*a*	*a*	*a*	*c*	*b*	*c*	*b*	*c*	*b*	*c*	*b*	*c*	*b*	*c*	*b*
10–12	1	0	1	0	0	1	1	2	2	*a*	*a*	*b*	*b*	*c*	*c*	*a*	*a*	*a*	*a*	*b*	*c*	*b*	*c*	*c*	*b*	*c*	*b*
13–15	1	0	1	1	1	2	2	0	0	*a*	*a*	*b*	*b*	*c*	*c*	*b*	*c*	*b*	*c*	*c*	*b*	*c*	*b*	*a*	*a*	*a*	*a*
16–18	1	0	1	2	2	0	0	1	1	*a*	*a*	*b*	*b*	*c*	*c*	*c*	*b*	*c*	*b*	*a*	*a*	*a*	*a*	*b*	*c*	*b*	*c*
19–21	2	0	2	0	1	0	2	1	2	*a*	*b*	*a*	*c*	*b*	*c*	*a*	*a*	*b*	*c*	*a*	*a*	*c*	*b*	*b*	*c*	*c*	*b*
22–24	2	0	2	1	2	1	0	2	0	*a*	*b*	*a*	*c*	*b*	*c*	*b*	*c*	*c*	*b*	*b*	*c*	*a*	*a*	*c*	*b*	*a*	*a*
25–27	2	0	2	2	0	2	1	0	1	*a*	*b*	*a*	*c*	*b*	*c*	*c*	*b*	*a*	*a*	*c*	*b*	*b*	*c*	*a*	*a*	*b*	*c*
28–30	3	1	0	0	2	2	1	1	0	*a*	*c*	*c*	*b*	*b*	*a*	*a*	*a*	*c*	*b*	*c*	*b*	*b*	*c*	*b*	*c*	*a*	*a*
31–33	3	1	0	1	0	0	2	2	1	*a*	*c*	*c*	*b*	*b*	*a*	*b*	*c*	*a*	*a*	*a*	*a*	*c*	*b*	*c*	*b*	*b*	*c.*
34–36	3	1	0	2	1	1	0	0	2	*a*	*c*	*c*	*b*	*b*	*a*	*c*	*b*	*b*	*c*	*b*	*c*	*a*	*a*	*a*	*a*	*c*	*b*
37–39	4	1	1	0	1	2	0	2	1	*a*	*b*	*c*	*a*	*c*	*b*	*a*	*a*	*b*	*c*	*c*	*b*	*a*	*a*	*c*	*b*	*b*	*c*
40–42	4	1	1	1	2	0	1	0	2	*a*	*b*	*c*	*a*	*c*	*b*	*b*	*c*	*c*	*b*	*a*	*a*	*b*	*c*	*a*	*a*	*c*	*b*
43–45	4	1	1	2	0	1	2	1	0	*a*	*b*	*c*	*a*	*c*	*b*	*c*	*b*	*a*	*a*	*b*	*c*	*c*	*b*	*b*	*c*	*a*	*a*
46–48	S	1	2	0	2	1	2	0	1	*a*	*c*	*b*	*c*	*a*	*b*	*a*	*a*	*c*	*b*	*b*	*c*	*c*	*b*	*a*	*a*	*b*	*c*
49–51	S	1	2	1	0	2	0	1	2	*a*	*c*	*b*	*c*	*a*	*b*	*b*	*c*	*a*	*a*	*c*	*b*	*a*	*a*	*b*	*c*	*c*	*b*
52–54	S	1	2	2	1	0	1	2	0	*a*	*c*	*b*	*c*	*a*	*b*	*c*	*b*	*b*	*c*	*a*	*a*	*b*	*c*	*c*	*b*	*a*	*a*

a Columns 1′,3,4,…,25, and 26 form OA_54_(6′ × 3^24^).

Columns 1,2,3,…,25, and 26 form OA_54_(2^1^ × 3^25^).

The entries **0,1,2,3,4,5,*a*,*b***, and ***c*** represent the column vectors (0,0,0), (1,1,1), (2,2,2), (3,3,3), (4,4,4), (5,5,5), (0,1,2), (1,2,0), and (2,0,1), respectively.
